# Intravenous injection of the oncolytic virus M1 awakens antitumor T cells and overcomes resistance to checkpoint blockade

**DOI:** 10.1038/s41419-020-03285-0

**Published:** 2020-12-12

**Authors:** Yang Liu, Jing Cai, Wenfeng Liu, Yuan Lin, Li Guo, Xincheng Liu, Zhen Qin, Cuiying Xu, Yanming Zhang, Xingwen Su, Kai Deng, Guangmei Yan, Jiankai Liang

**Affiliations:** 1 Department of Pharmacology, Zhongshan School of Medicine, Sun Yat-sen University, Guangzhou, 510080 China; 2 Department of Microbiology, Zhongshan School of Medicine, Sun Yat-sen University, Guangzhou, 510080 China; 3 Key Laboratory of Tropical Disease Control of Ministry of Education, Zhongshan School of Medicine, Sun Yat-sen University, Guangzhou, 510080 China

**Keywords:** Cancer microenvironment, Targeted therapies

## Abstract

Reversing the highly immunosuppressive tumor microenvironment (TME) is essential to achieve long-term efficacy with cancer immunotherapy. Despite the impressive clinical response to checkpoint blockade in multiple types of cancer, only a minority of patients benefit from this approach. Here, we report that the oncolytic virus M1 induces immunogenic tumor cell death and subsequently restores the ability of dendritic cells to prime antitumor T cells. Intravenous injection of M1 disrupts immune tolerance in the privileged TME, reprogramming immune-silent (cold) tumors into immune-inflamed (hot) tumors. M1 elicits potent CD8^+^ T cell-dependent therapeutic effects and establishes long-term antitumor immune memory in poorly immunogenic tumor models. Pretreatment with M1 sensitizes refractory tumors to subsequent checkpoint blockade by boosting T-cell recruitment and upregulating the expression of PD-L1. These findings reveal the antitumor immunological mechanism of the M1 virus and indicated that oncolytic viruses are ideal cotreatments for checkpoint blockade immunotherapy.

## Introduction

Cancer immunotherapy with immune checkpoint blockade (ICB) has made a breakthrough over the past decade^1–4^. Despite great efforts in this field, the majority of patients still fail to respond to this novel therapeutic regimen^5,6^. Effective cancer immunotherapy depends on the status of the immune response in the TME. High levels of preexisting tumor-infiltrating lymphocytes (TILs) and an inflammatory tumor transcriptional profile were identified as strong predictors of response to ICB^7,8^. Responsive tumors are usually considered immunologically “hot” tumors, while immunologically “cold” tumors do not benefit from ICB^9,10^. For instance, melanoma patients with higher numbers of CD8^+^ T cells in the TME were more likely to benefit from the PD-1 antibody pembrolizumab than those with lower numbers^11^. In addition, patients who respond to MPDL3280A, a PD-L1 antibody, had higher expression of PD-L1 and more IFN-γ secretion by tumor-infiltrating immune cells at baseline than nonresponders^12,13^. Hence, agents that overcome ICB resistance via the creation of immunologically “hot” tumors urgently need to be identified.

Oncolytic viruses (OVs) are naturally existing or genetically engineered viruses that represent ideal therapeutic platforms to treat cancer patients on the basis of the ability to selectively self-amplify in and kill tumor cells but leave normal cells intact^14^. Direct cell lysis has long been identified as the mechanism by which OVs kill tumor cells^15–17^. Until the recent decade, oncolytic virotherapy was considered a novel type of immunotherapy due to its capacities to modulate the TME and activate both innate and adaptive antitumor immune responses^18–20^. Since talimogene laherparepvec, which is derived from herpes simplex virus type 1 (HSV-1), became the first OV approved by the FDA for unresected melanoma, numerous clinical trials with OVs have been started^21–24^. Although many OVs appear to be effective antitumor agents with local administration, fewer studies have demonstrated the therapeutic efficacy of systemically administered OVs or the complete antitumor immune-activation process driven by OVs.

In 2014, our team identified M1, a strain of alphavirus isolated from culicine mosquitoes in the Hainan Province of China, as a novel OV capable of inducing endoplasmic reticulum (ER) stress-mediated apoptosis in zinc-finger antiviral protein-deficient cancer cells^25,26^. A series of small-molecule synergists have been found to magnify viral replication-mediated killing in vitro (cell lines), ex vivo (human tumor tissues), and in vivo (T cell-deficient nude mice)^27–30^. Relevant studies have shown that potent T-cell responses are required for the marked efficacy of cancer immunotherapy^9,11^; hence, T cells, especially cytotoxic CD8^+^ T cells, are probably critical to the efficacy of M1, although these cells may also mediate rapid viral elimination. Therefore, it is very important to explore the curative effect and antitumor immune mechanism of M1 in immunocompetent animal models.

Here, we report that intravenously administered M1 breaks local tolerance induces potent CD8^+^ T cell-dependent antitumor effects, and establishes systemic immune memory in poorly immunogenic tumor models. M1 induces immunogenic tumor cell death, subsequently activating dendritic cells (DCs) to initiate antitumor T-cell responses. More importantly, treatment with M1 overcomes resistance to PD-L1 antibody therapy. These findings highlight that the oncolytic virus M1 is a potential antitumor immunotherapeutic agent and the coordination of oncolytic virotherapy with ICB is an attractive strategy in the clinic.

## Materials and methods

### Cell lines

B16F10, RM-1, GL261, and BHK cell lines were purchased from the American Type Culture Collection, Shanghai Institute of Cell Biology, and Guangzhou Institute of Biomedicine and Health. Cells were cultured in DMEM, RPMI-1640, or DMEM F-12 supplemented with 10% (vol/vol) FBS and 1% penicillin/streptomycin (Life Technologies). All cells were cultured at 37 °C with 5% CO_2_.

### M1 virus production and quantification

M1 was provided by Guangzhou Virotech Pharmaceutical Technology Co., Ltd. M1-GFP is a recombinant derivative of M1 that expresses the jellyfish green fluorescent protein. Viral titers were determined by a TCID50 assay using BHK-21 cells and converted into pfu.

### In vivo experiments and tumor models

Animal studies were approved by the Animal Ethical and Welfare Committee of Sun Yat-sen University. The studies were randomized and blinded.

For evaluation of antitumor effects, 1 × 10^6^ B16F10 cells or 2.5 × 10^6^ RM-1 cells were inoculated s.c. into the hind flank of 6-week-old C57BL/6 mice. After palpable tumors developed (approximately 100 mm^3^), the mice were randomized to receive five doses of either the M1 virus (1 × 10^7^ pfu) or vehicle intravenously. Tumor length and width were measured, and tumor volume was calculated according to the formula (length × width^2^/2). Body weight was recorded. In total, 1 × 10^5^ GL261 cells were inoculated 2-mm deep in the top right of the lambdoidal suture in the brain of C57BL/6 mice on day 0. The mice were randomized to be injected with the vehicle or M1 virus (1 × 10^7^ pfu) intravenously daily on days 6–10 and days 18–22. At 148 days post initial tumor challenge, the five cured survivors and age-matched naive mice were subcutaneously challenged with 1 × 10^6^ GL261 cells in their flank. For combined therapy, B16F10 and RM-1 models were established as described above, and M1 (1 × 10^7^ pfu) or the vehicle was injected intravenously once per day for 5 days. Five doses (200 µg in 200 µl PBS per dose) of PD-L1 antibody (BE0101, BioXCell) or isotype antibody (BE0091, BioXCell) was administered intraperitoneally 48 h after the first M1 injection and then every 3 days.

For T-cell depletion experiments, B16F10 models were developed as described above. M1 (1 × 10^7^ pfu) was injected into the indicated mice daily on days 6–10, and a CD4 depletion antibody (BE0003-1, BioXCell), CD8 depletion antibody (BE0061, BioXCell) or isotype antibody (BE0090, BioXCell) was injected intraperitoneally on days 4, 7, 10, and 13 post tumor implantation.

### ATP secretion

The supernatant was collected and spun at 500 g for 5 min at 4 °C to remove cells and debris. ATP concentrations were measured with the ENLITEN^®^ ATP Assay System (Promega). ATP-driven chemiluminescence signals were recorded with a luminescence microplate reader (Synergy H1, BioTek).

### CRT exposure

Cells were stained with a rabbit CRT APC-conjugated antibody (bs-6913R-APC, Bioss) on ice for 30 min. After washing with PBS, all samples were analyzed by flow cytometry (CytoFLEX, Beckman Coulter).

### Cell viability assays

After infection, 3-(4,5-dimethylthiazol-2-yl)-2,5-diphenyltetrazolium bromide (MTT) was added to each well (1 mg/ml final concentration), and the cells were incubated at 37 °C for another 3 h. The medium was removed, and the MTT precipitate was dissolved in 500 μl dimethyl sulfoxide. The absorbance was determined at 490 nm using a microplate reader (Synergy H1, BioTek).

### BMDC and T-cell isolation

Bone marrow cells were collected from the tibias and femurs of C57BL/6 mice and cultured with complete RPMI-1640 medium containing 20 ng/ml mGM-CSF (PeproTech). Fresh medium supplemented with mGM-CSF was added to the culture 3 days later. Immature BMDCs were collected and ready to use on day 7. Naive T cells were isolated from the spleen with a negative CD3^+^ T-cell isolation kit (STEMCELL Technologies).

### ELISA

Cell culture supernatants were obtained. The concentrations of IL-12, IL-1β, TNF-α, granzyme B, IFN-γ (Multi Sciences), and perforin (Elabscience) were measured with ELISA kits.

### Flow cytometry

Tumors tissues were harvested, and single-cell homogenates were prepared by using a mouse tumor dissociation kit (Miltenyi Biotec) and passed through a 70-mm strainer before the red blood cells were lysed. Surface markers were stained with the following antibodies at 4 °C for 30 min: CD45-BV510 (563891, BD), CD3-PerCP-Cy5.5 (551163, BD), CD4-FITC (100406, BioLegend), CD8-Alexa Fluor 700 (557959, BD), CD69-PE/Cy7 (104512, BioLegend), CD44-BV650 (103049, BioLegend), PD-1-PE-CF594 (562523, BD) and PD-L1-PE (558091, BD). For forkhead box P3 (FOXP3) staining, a transcription factor buffer set (00-5523-00, eBioscience) was used.

For DCs staining, following antibodies were used: CD11c-APC (17-0114-82, eBioscience), MHC I-PE (12-5958-82, eBioscience) and CD86-eFluor 450 (48-0862-82, eBioscience).

### qRT-PCR

RNA was extracted with TRIzol reagent (Thermo Fisher), and reverse transcription was performed with oligo dT and RevertAid Reverse Transcriptase (Thermo Fisher). Quantitative PCR was performed with SuperReal PreMix SYBR Green (TIANGEN) in the Applied Biosystems 7500 Fast Real-Time PCR System (Life Technologies). The gene expression levels were normalized to those of β-actin. The amplification primers were purchased from GeneCopoeia.

### Lymphocytotoxicity test

TILs were isolated from tumors with a tumor lymphocyte infiltration kit (Solarbio). TDLN and splenic lymphocytes were isolated from the TDLN and spleen with lymphocyte separation fluid (Dakewe). Lymphocytes were cocultured with preseeded B16F10 cells for 2 days. The cells were washed with PBS twice to remove suspended lymphocytes, and the viability of adherent tumor cells was detected by MTT assays as described above. Lymphocytotoxicity was calculated with the following formula: lymphocytotoxicity = (A_tumor cells_ − A_tumor cells+lymphocytes_)/(A_tumor cells_ − A_blank_) × 100%.

### Statistical analysis

All statistical analyses were performed using GraphPad Prism 6.0 and 8.0. All sample sizes and statistical methods are indicated in the corresponding figure legend. No statistical methods were used to predetermine the sample size. No generated data were excluded. If the data were normally distributed (as determined by the Shapiro–Wilk test) and homoscedastic (as determined by Bartlett’s test), Student’s *t* test (for two groups) and one-way analysis of variance (ANOVA) (more than two groups) were used to test the mean difference. Most of the data were analyzed by Student’s *t* test or one-way ANOVA with Dunnett’s tests for multiple comparisons. Tumor volumes were analyzed by Student’s *t* test or one-way ANOVA of the final time point as indicated in graphs. Survival was analyzed by the Kaplan–Meier method and compared using the log-rank test. Bars show the mean ± SD. Significant differences were accepted if the *P* value was <0.05.

## Results

### Intravenous injection of M1 elicits therapeutic antitumor effects and establishes long-term protective immune memory in vivo

**Fig. 1:**
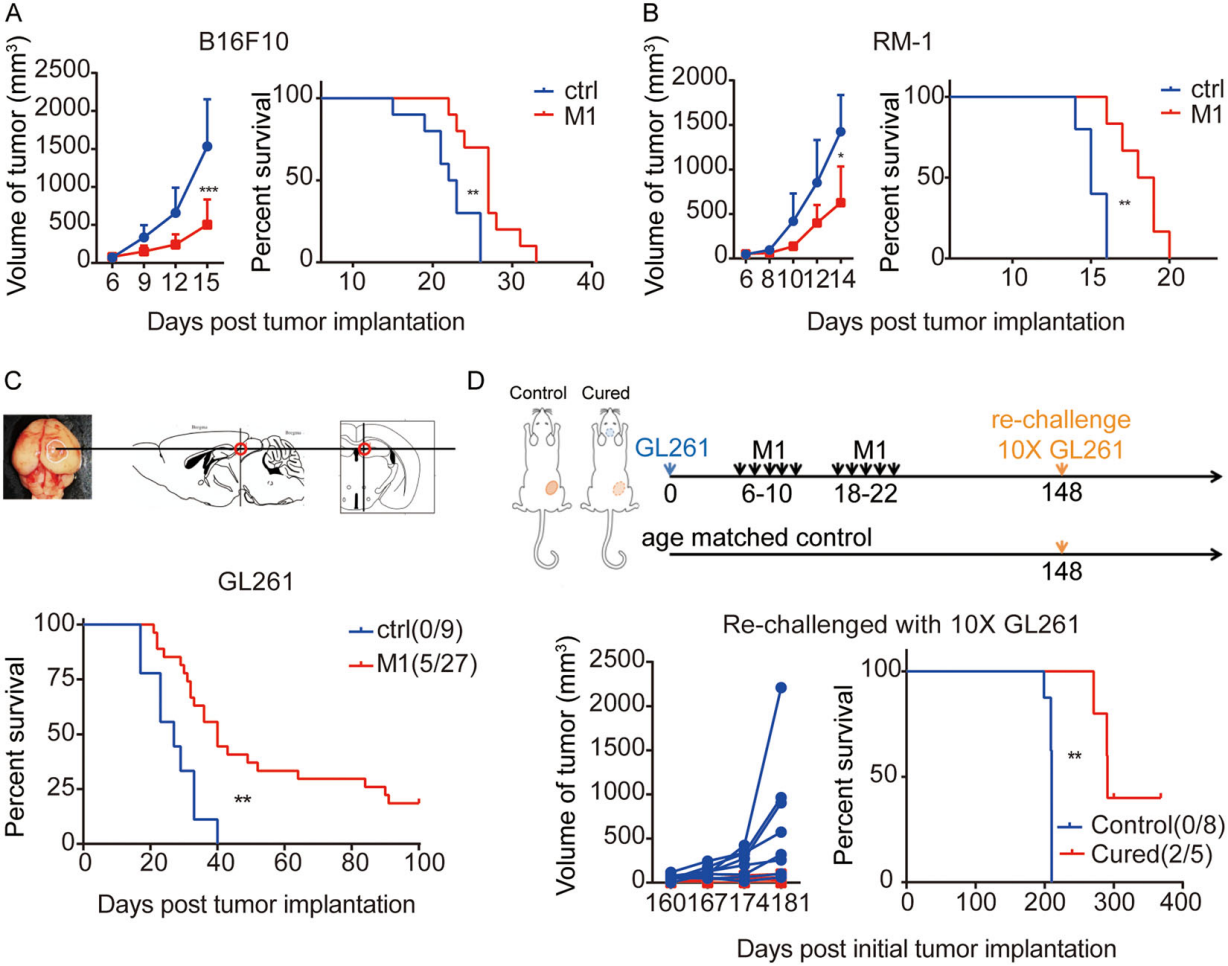
Intravenous injection of M1 virus produces an antitumor therapeutic effect and establishes long-term protective immune memory in vivo. **A** C57BL/6J mice were implanted subcutaneously in the right flank with B16F10 cells on day 0 and treated intravenously with vehicle (ctrl) (*n* = 10) or M1 (*n* = 10) (1 × 10^7^ plaque-forming units (pfu)) once per day on days 6–10. Tumor growth curves (left) and Kaplan–Meier survival curves (right) are shown. *P* values were determined by Student’s *t* test of the final time point as indicated in graphs and log-rank test. **B** C57BL/6J mice were implanted subcutaneously in the right flank with RM-1 cells at day 0 and treated intravenously with ctrl (*n* = 5) or M1 (*n* = 6) (1 × 10^7^ pfu) daily on days 6–10. Tumor growth curves (left) and Kaplan–Meier survival curves (right) are shown. *P* values were determined by Student’s *t* test analysis of the final time point as indicated in the graphs and by the log-rank test. **C** Diagrammatic sketch of the mouse orthotopic glioma model. GL261 cells were inoculated 2 mm deep in the top right of the lambdoidal suture of the brain on day 0. The mice were injected with ctrl or M1 virus (1 × 10^7^ pfu) via the tail vein once per day on days 6–10 and days 18–22. Long-term survivors (>100 days) are indicated. Kaplan–Meier survival curves are shown. The *P* value was determined by the log-rank test. **D** Diagrammatic sketch of the subcutaneous tumor rechallenge model. Five long-term survivors (cured) were obtained from the M1-treated groups. **C** At 148 days post initial tumor challenge, the five cured survivors were rechallenged with a ten times larger number of GL261 cells than the number first used by subcutaneous implantation in the flank. Age-matched naïve mice (*n* = 8) implanted with an equivalent number of GL261 tumor cells served as controls. The rechallenge tumor growth curves (left) and Kaplan–Meier survival curves (right) are shown. The mice that completely rejected the tumor rechallenge are indicated. The *P* value was determined by the log-rank test. Data are shown as the mean ± SD. *P < 0.05; **P < 0.01; ***P < 0.001. See also Supplementary Fig. S1.

To explore the therapeutic potency of M1 in immunocompetent mice, we established three poorly immunogenic tumor models, including subcutaneous B16F10 skin melanoma model, subcutaneous RM-1 prostatic carcinoma model, and orthotopic GL261 glioma model in immunocompetent mice. The animals were randomized to receive an intravenous injection of either the M1 virus or vehicle control. M1 treatment produced potent tumor growth inhibition and improved survival in these three models (Fig. 1A–C and Supplementary Fig. S1A). There were no signs of toxicity or weight loss during the whole experiment (Supplementary Fig. S1B–D). Notably, five long-term survivors were found in the GL261 glioma model after M1 treatment, indicating complete tumor regression (Fig. 1C). These cured survivors were utilized to check whether M1 virotherapy elicited antitumor immune memory. At 148 days post initial tumor challenge, the five cured survivors were rechallenged with ten times more GL261 cells than was first implanted subcutaneously in the flank. Age-matched naive mice implanted with an equivalent number of GL261 cells served as controls (Fig. 1D). All age-matched naive mice showed rapid tumor growth and died due to the tumor burden. Conversely, three long-term survivors had slow tumorigenesis, and two mice had completely tumor-free survival (Fig. 1D). This demonstrates that systemic immunological surveillance to prevent relapse metastases and recurrences develops after M1 virotherapy, which is the ultimate goal of successful cancer immunotherapy.

### M1 induces immunogenic cell death (ICD) and endows tumors with an inflammatory gene signature

**Fig. 2:**
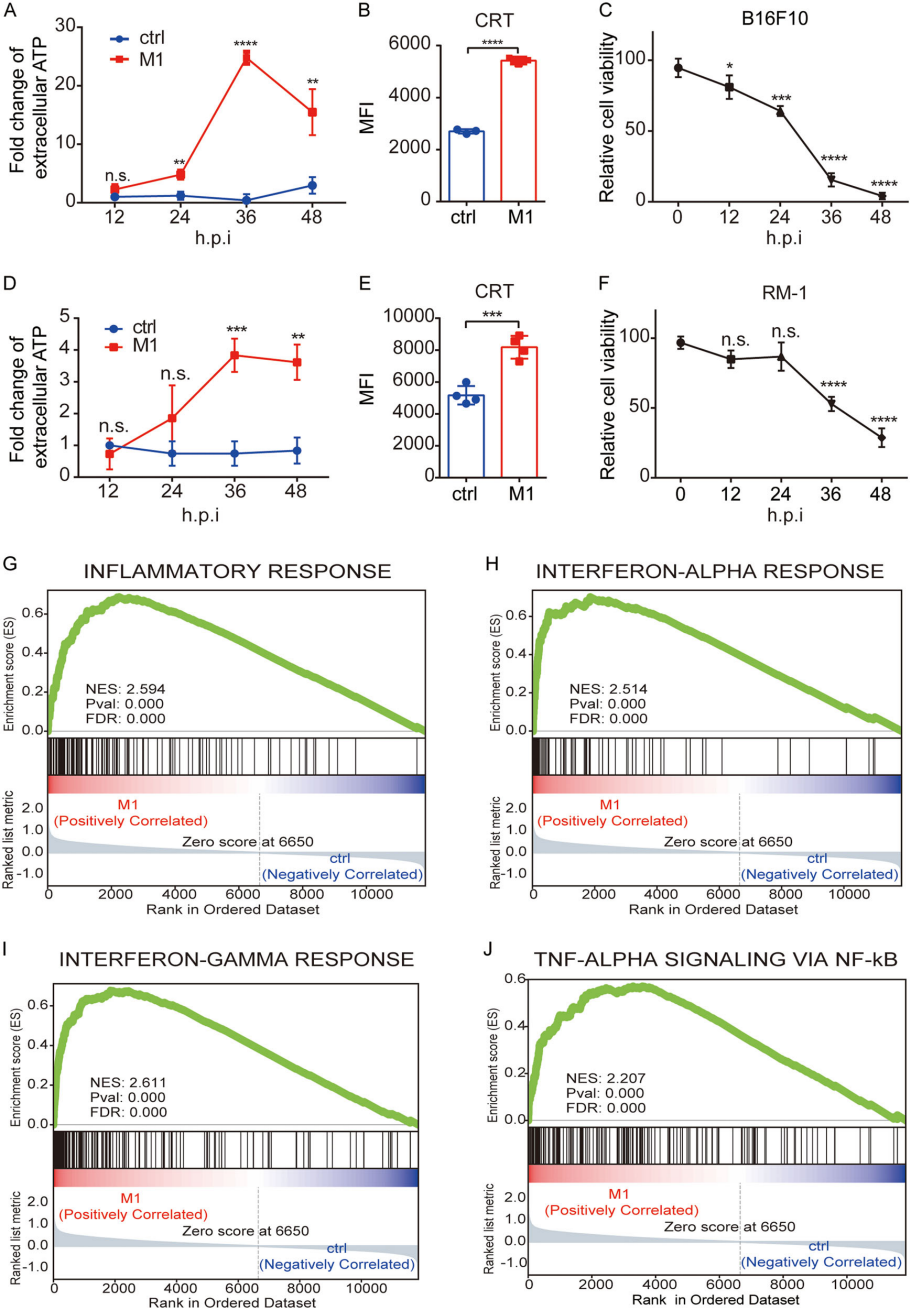
M1 induces ICD and endows tumors with an inflammatory gene signature. **A** The B16F10 cell line was treated with ctrl or M1 (multiplicity of infection (MOI) = 1), and ATP secretion into the supernatant, which was collected at the indicated hours post infection (h.p.i), was detected with an ATP detection kit. *n* = 3. *P* values were determined by a two-tailed Student’s *t* test. **B** The B16F10 cell line was treated with ctrl or M1 (MOI = 1) for 24 h, and CRT exposure was examined by flow cytometry. *n* = 3. *P* value was determined by a two-tailed Student’s *t* test. **C** B16F10 cells were treated with M1 (MOI = 1), and relative cell viability was determined by an MTT assay at the indicated h.p.i. *n* = 3. Following one-way ANOVA, Dunnett’s multiple comparisons test was performed to compare the mean of each group with the mean of the 0 h.p.i groups. **D** RM-1 cells were treated with ctrl or M1 (MOI = 1), and ATP secretion into the supernatant, which was collected at the indicated h.p.i., was detected with an ATP detection kit. *n* = 3. The *P* value was determined by a two-tailed Student’s *t* test. **E** RM-1 cells were treated with ctrl or M1 (MOI = 1) for 36 h, and CRT exposure was examined by flow cytometry. *n* = 4. The *P* value was determined by a two-tailed Student’s *t* test. **F** RM-1 cells were treated with M1(MOI = 1), and relative cell viability was determined by an MTT assay at the indicated h.p.i. *n* = 3. Following one-way ANOVA, Dunnett’s multiple comparisons test was performed to compare the mean of each group with the mean of the 0 h.p.i groups. **G**–**J** C57BL/6 mice were implanted subcutaneously in the right flank with B16F10 cells on day 0 and treated intravenously with ctrl (*n* = 4) or M1 (*n* = 4) (1 × 10^7^ pfu) once per day on days 6–10. The tumors were harvested on day 12, the total RNA was extracted, and RNA sequencing was then performed. GSEA results for the inflammatory response (**G**), IFN-αresponse (**H**), IFN-γ response (**I**), and TNF-α response (**J**) are shown. MFI mean fluorescence intensity. Data are reported as the mean ± SD. n.s. not significant; *P < 0.05; **P < 0.01; ***P < 0.001; ****P < 0.0001. See also Supplementary Fig. S2.

We previously reported that M1 triggers prolonged and severe ER stress in susceptible cancer cells^26^. Activation of ER stress confers immunogenic characteristics to tumor cells because ER stress can orchestrate the danger signaling pathways responsible for the trafficking of vital damage-associated molecular patterns (DAMPs) and subsequent immune responses^31,32^. In the early stage of ICD, cytomembrane-exposed calreticulin (CRT) and secreted adenosine triphosphate (ATP) are crucial DAMPs^33,34^. First, we tested whether M1 infection promotes the release of these DAMPs in vitro. The level of ATP was elevated at 24 h post infection and reached a peak at 36 h in M1-treated B16F10 cell supernatants (Fig. 2A). The exposure of CRT was also increased at 24 h (Fig. 2B). Similar phenomena were also found with the RM-1 cell line (Fig. 2D, E). These data indicate that M1 induces the release of DAMPs during tumor cell killing (Fig. 2C, F), representing ICD.

With this favorable “danger” backdrop, we then asked whether M1 can act as an immunostimulatory agent and convert the status of the TME. B16F10 melanoma is recognized as an immune-desert tumor. We established a B16F10 subcutaneous model in immunocompetent C57BL/6 mice and treated them with M1 virus intravenously, followed by RNA sequencing of the tumors. Gene set enrichment analysis (GSEA) revealed that upon M1 treatment, the expression of apoptosis- and inflammation-associated genes was largely upregulated (Supplementary Fig. S2a, g), suggesting that M1 killed tumor cells and triggered an inflammatory response. GSEA also revealed that the DNA repair pathway was downregulated in M1-treated tumors, which was consistent with our previous finding that M1 induces DNA damage that leads to tumor cell apoptosis (Supplementary Fig. S2B). Additionally, gene sets indicating the release of immune-boosting cytokines such as interferon (IFN)-α, IFN-γ, and tumor necrosis factor (TNF)-α (Fig. 2H–J) were apparent in the M1-treated tumors, providing potential proinflammatory signals to generate antitumor immunity. All the results described above demonstrate that M1 induces ICD and shapes the TME into an inflammatory state.

### Tumor cells lysed by M1 mature iDCs, which allowed priming of de novo T-cell responses ex vivo

**Fig. 3:**
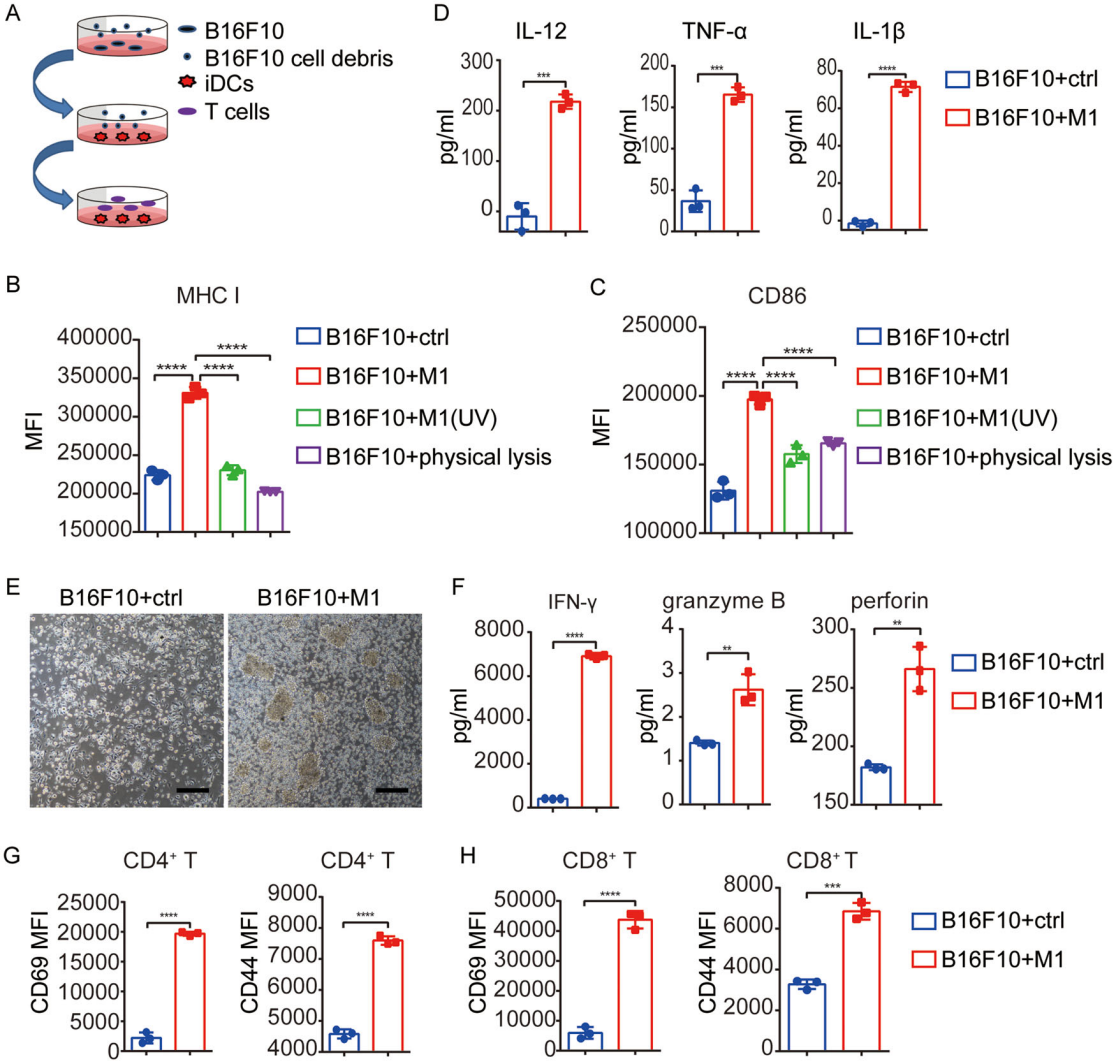
Tumor cells lysed by M1 mature iDCs and enable the iDCs to prime de novo T-cell responses ex vivo. **A** Diagrammatic sketch of the coculture experiment performed ex vivo. B16F10 cells were treated with ctrl, M1, UV-inactivated M1, or physical lysis on day 0, and then the supernatant of each group was collected and fed to immature DCs (iDCs) on day 2. One batch of DCs was used to detect maturation markers by flow cytometry and secreted cytokines by ELISA on day 4. The other batch of DCs was cocultured with naive T cells on day 4, and T-cell activation was evaluated on day 8. **B**, **C** MHC I (**B**) and CD86 (**C**) expression on DCs fed the indicated supernatants was detected by flow cytometry on day 4. *n* = 3. *P* values were determined by one-way ANOVA with Dunnett’s tests for multiple comparisons. **D** IL-12, TNF-α, and IL-1β levels in the culture medium of DCs fed with the indicated supernatants were determined by ELISA on day 4. *n* = 3. The *P* value was determined by a two-tailed Student’s *t* test. **E** Phase-contrast images of proliferating T-cell clones on day 8 are shown. The images are representative of three independent experiments. Scale bars, 50 μm. F IFN-γ, granzyme B, and perforin concentrations were determined by ELISA in the DC plus T-cell coculture system on day 8. *n* = 3. The *P* value was determined by a two-tailed Student’s *t* test. G-H, CD69, and CD44 expression on CD4^+^ T cells (**G**) and CD8^+^ T cells (**H**) was evaluated on T cells in the DC plus T-cell coculture system on day 8. *n* = 3. The *P* value was determined by a two-tailed Student’s *t* test. Data are shown as the mean ± SD. **P < 0.01; ***P < 0.001; ****P < 0.0001. See also in Supplementary Figs. S3 and S4.

We assumed that many PAMPs, DAMPs, and tumor-associated antigens (TAAs) would emerge with M1-mediated killing of tumor cells and these contents may potentiate DC phenotypic maturation. Therefore, B16F10 cells were treated with M1, UV-inactivated M1, or physically lysed by repeated freeze-thaw cycles. Then, we fed iDCs separately with the supernatant collected from each processing group and detected maturation markers on the DC surface 48 h later (Fig. 3A and Supplementary Fig. S3A and B). Compared with the control, the B16F10 oncolysate generated with M1 significantly upregulated the expression of MHC I on DCs, unlike the supernatants obtained from M1(UV)-infected or physically lysed B16F10 cells (Fig. 3B). DCs exhibited modestly increased expression of the costimulatory molecule CD86 after feeding with the supernatants from M1(UV)-infected or physically lysed B16F10 cells, but these levels were far lower than those DCs fed the M1-infected cell supernatant (Fig. 3C). In addition, the DCs supplied with the supernatant from M1-infected tumor cells produced much higher concentrations of IL-12, TNF-α, and IL-1β (Fig. 3D). These cytokines were derived from the DCs, not the tumor cells supernatants (Supplementary Fig. S4A–C).

To determine whether these activated DCs can prime de novo T-cell responses effectively ex vivo, we stimulated iDCs with supernatants from M1-infected B16F10 cells and then cocultured these DCs with naive T cells (Fig. 3A). More and denser proliferating T-cell clones were observed in the wells where T cells were cocultured with DCs stimulated with supernatants from M1-infected B16F10 cells (Fig. 3E). Higher levels of IFN-γ, granzyme B, and perforin were detected in the culture medium from wells where T cells were cocultured with DCs stimulated with supernatants from M1-infected B16F10 cells (Fig. 3F), indicating the activation and increased cytotoxic potential of the T cells. Furthermore, both CD4^+^ T cells and CD8^+^ T cells were primed by DCs stimulated with supernatants from M1-infected B16F10 cells, as demonstrated by the elevated expression of the activation markers CD69 and CD44 (Fig. 3G, H). All the evidence above illustrated that oncolysates produced by M1 infection effectively restored the ability of DCs to drive effector T-cell generation ex vivo.

### M1 acts as an immunostimulatory agent and induces T-cell recruitment and activation in TME

**Fig. 4:**
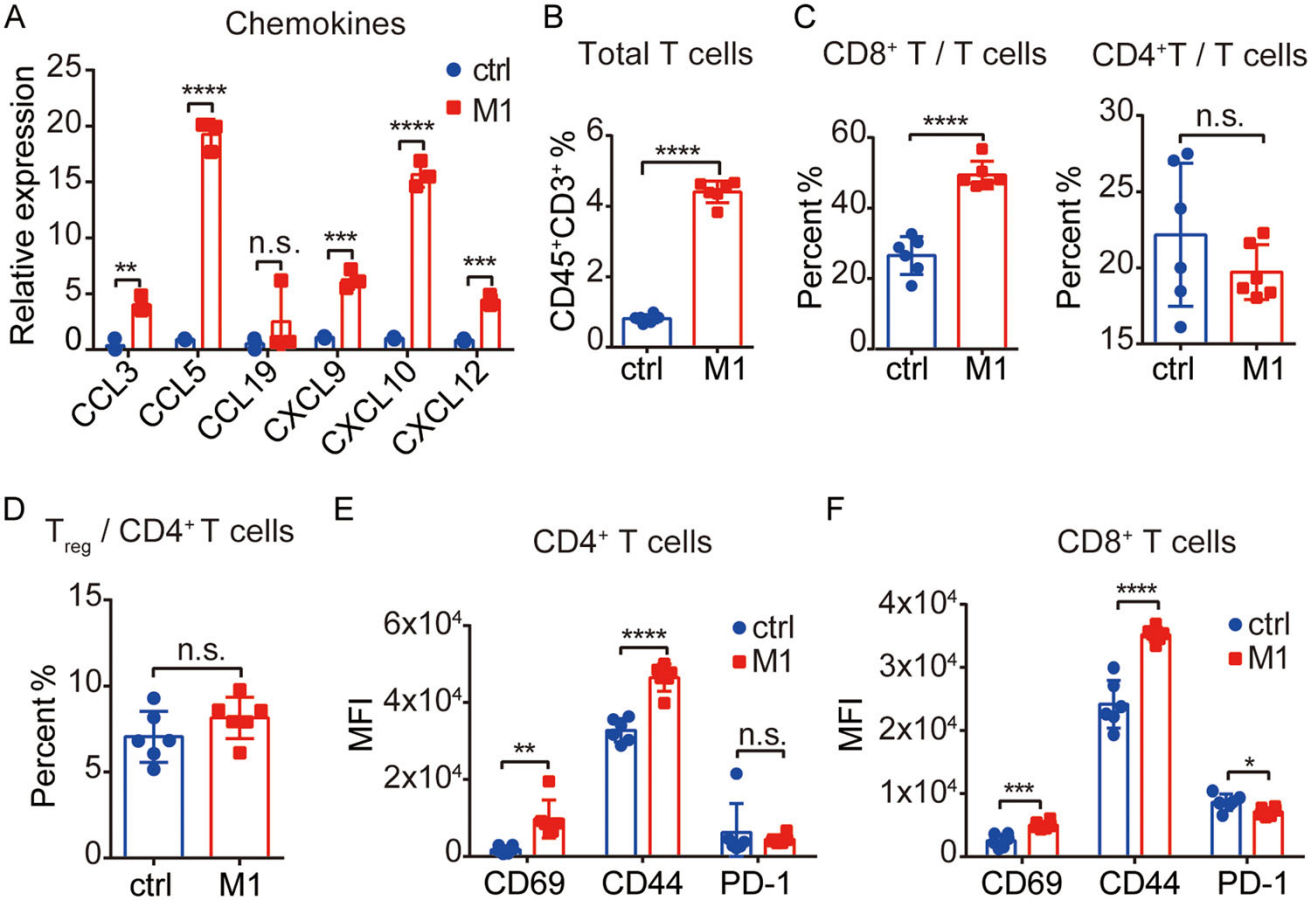
M1 acts as an immunostimulatory agent and induces T-cell recruitment and activation in tumor microenvironment (TME). **A** C57BL/6 mice were implanted subcutaneously in the right flank with B16F10 cells on day 0 and treated intravenously with ctrl (*n* = 3) or M1 (*n* = 3) (1 × 10^7^ pfu) once per day on days 6–8. The tumors were harvested on day 9, the total RNA was isolated, and qRT-PCR was performed to detect the expression of chemokines. The *P* value was determined by a two-tailed Student’s *t* test. **B**–**D** C57BL/6 mice were implanted subcutaneously in the right flank with B16F10 cells on day 0 and treated intravenously with ctrl (*n* = 6) or M1 (*n* = 6) (1 × 10^7^ pfu) once per day on days 6–10. The tumors were harvested on day 13 and analyzed by flow cytometry to assess intratumoral T cells (**B**), the percentages of CD8^+^ and CD4^+^ T cells in the total T-cell population (**C**) and T_reg_ in the CD4^+^ T-cell population (**D**). The *P* value was determined by a two-tailed Student’s *t* test. **E**, **F** the expression of CD69, CD44, and PD-1 on CD4^+^ T cells (**E**) and CD8^+^ T cells (F) gated from **B** to **D** was detected by flow cytometry. The *P* value was determined by a two-tailed Student’s *t* test. Data are shown as the mean ± SD. n.s. not significant; *P < 0.05; **P < 0.01; ***P < 0.001; ****P < 0.0001.

To confirm that M1 drives robust T-cell immunity in vivo, we first checked whether M1 virotherapy creates an immunosupportive chemokine milieu within tumor tissue. Chemokine (C–C motif) ligand (CCL) family chemokines, such as CCL3 and CCL5, and chemokine (C–X–C motif) ligand (CXCL) family chemokines, such as CXCL9, CXCL10, and CXCL12, have been shown to promote the recruitment of T cells to sites of inflammation or infection^35^; these chemokines showed significant increases in mRNA levels in tumor tissues from M1-injected mice (Fig. 4A). The same trend (approximately fivefold increase) was observed for T-cell infiltration into the TME (Fig. 4B). Cytotoxic CD8^+^ T cells are the main tumor executioners. Among T cells, the number of CD8^+^ T cells in animals treated with M1 was nearly twice that in control animals, while the ratio of CD4^+^ T cells showed no difference (Fig. 4C). Regulatory T cells (T_reg_) are a well-known immunosuppressive subpopulation of CD4^+^ T cells, and some other oncolytic virus monotherapies lead to the accumulation of intratumoral T_reg_^36,37^. However, we did not find that CD4^+^ T cells polarized in the T_reg_ direction after M1 treatment (Fig. 4D). The expression of the activation markers CD69 and CD44 was substantially upregulated on CD4^+^ and CD8^+^ tumor-infiltrating T cells in M1-treated mice (Fig. 4E, F), indicating the reinvigoration of T-cell populations. Modest but significant downregulation of PD-1 expression was found on CD8^+^ T cells after M1 treatment, while PD-1 expression on CD4^+^ T cells remained unchanged (Fig. 4E, F). Overall, these changes revealed that M1 therapy triggered an influx and activation of T cells in the TME, especially CD8^+^ T lymphocytes.

### The M1 virus induces local and systemic CD8+ T cell-dependent antitumor immunity

**Fig. 5:**
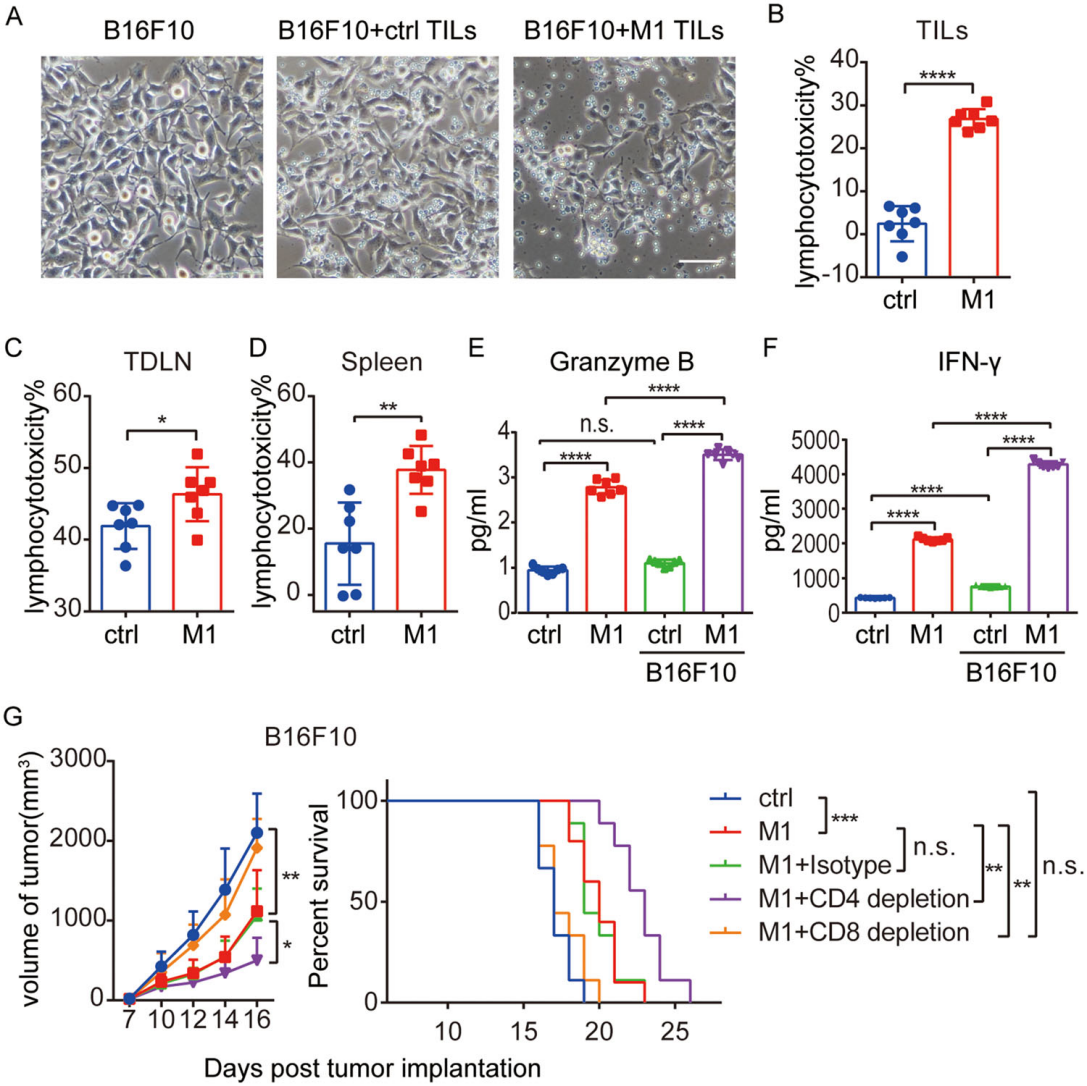
The M1 virus induces local and systemic CD8^+^ T cell-dependent antitumor immunity. **A** Tumor-infiltrating lymphocytes (TILs) were isolated from B16F10 tumor tissue samples and cocultured with the B16F10 cell line for 48 h; phase-contrast images are shown. The images are representative of six biological repetitions. Scale bars: 50 μm. **B**–**D** Lymphocytes isolated from B16F10 tumor tissue samples (TILs), tumor-draining lymph nodes (TDLN), and the spleen were cocultured with B16F10 cells for 48 h to evaluate lymphocytotoxicity. *n* = 7. The *P* value was determined by a two-tailed Student’s *t* test. **E**, **F** Granzyme B (**E**) and IFN-γ (**F**) were analyzed in supernatants of splenic lymphocytes cocultured with or without B16F10 cells. *n* = 6. The *P* value was determined by one-way ANOVA with Dunnett’s test. **G** C57BL/6 mice were implanted subcutaneously in the right flank with B16F10 cells on day 0 and treated intravenously with ctrl (*n* = 6) or M1 (*n* = 6) (1 × 10^7^ pfu) once per day on days 6–10. An anti-CD4 depletion antibody, anti-CD8 depletion antibody, or isotype control antibody was injected intraperitoneally on days 4, 7, 10, and 13. B16F10 tumor growth (left) and Kaplan–Meier survival (right) curves for the mice in each group are shown. ctrl, *n* = 9; M1, *n* = 10; M1 + isotype, *n* = 9; M1 + CD4 depletion, *n* = 9; M1 + CD8 depletion, *n* = 9. *P* values were determined by one-way ANOVA of the final time point as indicated in the graphs and by the log-rank test. Data are shown as the mean ± SD. n.s., not significant; *P < 0.05; **P < 0.01; ***P < 0.001. ****P < 0.0001. See also Supplementary Figs. S5 and S6.

To further investigate whether T cells activated by M1 treatment are able to kill tumor cells, TILs enriched from B16F10 tumors were cocultured with B16F10 cells, and the lymphocytotoxicity of the TILs was determined by measuring the viability of adhered tumor cells. The tumor cells cocultured with TILs from untreated mice had a similar morphology and growth density to those cultured under normal conditions, suggesting the exhaustion and disruption function of lymphocytes in the TME (Fig. 5A). However, B16F10 cells were markedly killed by TILs from mice receiving M1 therapy (Fig. 5A). We ruled out the possibility of direct virus killing by confirming no viral particles could be detected among the TILs from the mice receiving M1 therapy (Supplementary Fig. S5A). Lymphocytotoxicity of the TILs in both groups was calculated (Fig. 5B). In addition to local lymphocyte activation, systemic antitumor immunity was also investigated by using lymphocytes isolated from the tumor-draining lymph node (TDLN) and spleen. M1 injection improved the lymphocytotoxicity of both splenic and TDLN lymphocytes (Fig. 5C, D). Enzyme-linked immunosorbent assay (ELISA) revealed that M1 therapy augmented granzyme B and IFN-γ secretion by splenic lymphocytes (Fig. 5E, F). Furthermore, splenic lymphocytes from the M1 group released more granzyme B upon reencounter of B16F10 cells, while the lymphocytes from the control group remaining unresponsive (Fig. 5E). ELISA analysis of IFN-γ also showed a stronger response against B16F10 cells by the splenic lymphocytes harvested from the M1 group than those from the control group (Fig. 5F). These results demonstrate that M1 potently awakens local and systemic antitumor T-cell immunity in tumor-bearing mice.

Because both CD4^+^ and CD8^+^ T cells were activated ex vivo and in vivo, depletion antibodies specific for CD4^+^ or CD8^+^ T cells were used to explore their roles in M1-induced antitumor effects. Flow cytometry analysis confirmed the specific loss of each type of T cell in the TME, spleen, and peripheral blood with the indicated depletion antibody (Supplementary Fig. S6A–C). CD8^+^ T-cell depletion completely abrogated the therapeutic effect and survival benefit induced by M1 (Fig. 5G). Intriguingly, CD4^+^ T cell depletion resulted in a better antitumor effect (Fig. 5G). The results described above demonstrate that M1 elicits a CD8^+^ T cell-dependent antitumor therapeutic effect.

### M1 induces PD-L1 upregulation and overcomes PD-L1 blockade resistance

**Fig. 6:**
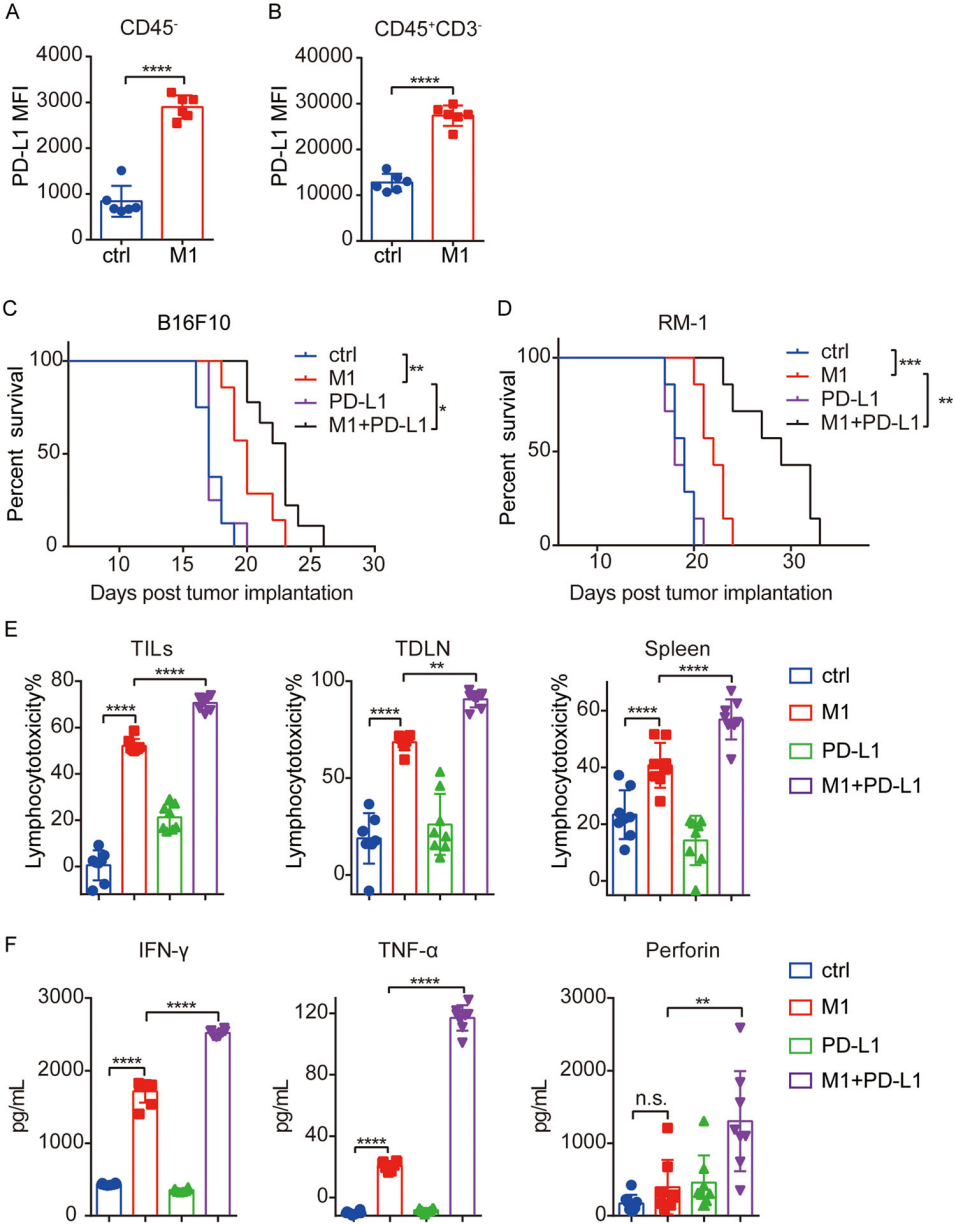
M1 induces PD-L1 upregulation and overcomes PD-L1 blockade resistance. **A**, **B** C57BL/6 mice were implanted subcutaneously in the right flank with B16F10 cells on day 0 and treated intravenously with ctrl (*n* = 6) or M1 (*n* = 6) (1 × 10^7^ pfu) once per day on days 6–10. The expression of PD-L1 on CD45^−^ and CD45^+^ CD3^−^ cells in tumor tissue samples were detected by flow cytometry on day 13. The *P* value was determined by a two-tailed Student’s *t* test. **C** C57BL/6 mice were implanted subcutaneously in the right flank with B16F10 cells on day 0 and treated intravenously with ctrl or M1 (1 × 10^7^ pfu) daily on days 7–11. A PD-L1 antibody or isotype control antibody was injected intraperitoneally on days 9, 12, 15, 18, and 21. Kaplan–Meier survival curves (right) are shown. ctrl, *n* = 8; M1, *n* = 7; PD-L1, *n* = 8; M1 + PD-L1, *n* = 9. The *P* value was determined by the log-rank test. **D** C57BL/6 mice were implanted subcutaneously in the right flank with RM-1 cells on day 0 and treated intravenously with ctrl or M1 (1 × 10^7^ pfu) once per day on days 6–10. The PD-L1 antibody or isotype control was injected intraperitoneally on days 8, 11, 14, 17, and 20. Kaplan–Meier survival curves (right) are shown. *n* = 7. The *P* value was determined by the log-rank test. **E** Tumor models were established, and M1 plus PD-L1 antibody treatment was conducted as shown in (**C**). Lymphocytes isolated from B16F10 tumor tissue samples (TILs), tumor-draining lymph nodes (TDLN), and the spleen on day 15 were cocultured with B16F10 cells for 48 h to evaluate cytotoxicity. *n* = 8. The *P* value was determined by one-way ANOVA with Dunnett’s test. **F** IFN-γ, TNF-a, and perforin were analyzed in supernatants of splenic lymphocytes cocultured with B16F10 cells. *n* = 8. The *P* value was determined by one-way ANOVA with Dunnett’s test. Data are shown as the mean ± SD. *P < 0.05; **P < 0.01; ***P < 0.001. ****P < 0.0001. See also Supplementary Fig. S7.

IFN-γ is a cytokine that plays a pivotal role in host antitumor immunity. However, in recent years, IFN-γ has been reported to induce PD-L1 expression, which counteracts the immune response^38^. Since M1 treatment increased the secretion of IFN-γ by lymphocytes (Fig. 5F), we evaluated the expression of PD-L1 in the TME. PD-L1 expression was significantly increased on tumor cells and some immune cells after M1 treatment, as indicated by flow cytometry analysis of PD-L1 expression on CD45^−^ (Fig. 6A) and CD45^+^ CD3^-^ cells (Fig. 6B). However, high PD-L1 expression is one of the determinants of favorable clinical responsiveness to anti-PD-L1 therapy^12,39^. Thus, these findings strongly suggest that blocking the PD-1-PD-L1 interaction in the context of M1 treatment may yield additional antitumor effects. A PD-L1 antibody alone did not result in tumor growth inhibition in the B16F10 model or RM-1 model, whereas the combination of the PD-L1 antibody with M1 showed a significant delay in tumor growth and survival extension (Fig. 6C, D). There were no signs of toxicity or weight loss during the whole experiment (Supplementary Fig. S7A, B). TILs, TDLN’s and splenic lymphocytes from mice that received combined therapy showed stronger cytotoxicity against B16F10 cells than those who received monotherapy or control treatment (Fig. 6E). ELISA analysis of IFN-γ, TNF-α, and perforin concentrations also showed the highest secretion was performed by splenic lymphocytes harvested from mice who received combined therapy after reencounter of B16F10 cells (Fig. 6F). These observations indicate that M1 therapy can be augmented by anti-PD-L1 therapy and overcome PD-L1 blockade resistance.

## Discussion

In this study, we show that intravenous administration of the M1 virus breaks an immune-privileged TME. The tumor death process mediated by M1 is adjuvant and antigenic, providing several signals to the immune system. First, extensive replication of M1 in tumor cells leads to the release of viral nucleic acids and protein components, which serve as PAMPs (signal 1). Additionally, M1-mediated cell death leads to the emergence of potent danger signals, including sundry DAMPs (signal 2) and the exposure of TAAs in the context of MHC molecules (signal 3) on DCs. Then, viruses often induce inflammation and stimulate the production of chemokines and cytokines (signal 4) that attract T cells to the TME and activate these cells. These four signals, together with costimulatory molecules on DCs (signal 5), produce potent antitumor T-cell responses. Based on our previous and present results, M1 virotherapy is versatile immunotherapy due to its therapeutic activity mediated by combining direct in situ tumor killing with the ability to immunologically inflame tumors and then initiate powerful antitumor immunity.

M1 treatment brings in CD8^+^ T cell-dependent antitumor effects; therefore, any approach that seeks to mobilize or manipulate CD8^+^ T cells is likely to be a promising strategy for synergy with M1. However, the divergent role of CD4^+^ T cells may be explained by the depletion of suppressive T_reg_ cells even though these cells did not polarize into T_reg_ direction after M1 treatment. Hence, potentiating M1-induced antitumor immunity by modulating T_reg_ cells is our potential research interest.

ICB has revolutionized cancer treatment. In general, cancers responsive to ICB have a higher mutational burden and more neoantigens than nonresponsive cancers^40,41^. Unfortunately, many patients have a low frequency of TAA-reactive T cells and respond poorly to ICB due to a paucity of antigen presentation by APCs and the weak immunogenicity of tumor cells. Selective destruction of neoplastic tissues by OVs may lead to antigen-agnostic boosting of neoantigen-specific cytotoxic T-lymphocyte responses. Furthermore, the powerful capability to recruit T cells into the TME makes M1 an ideal cotreatment for checkpoint blockade therapy. On the other hand, upregulation of PD-L1 expression after M1 treatment makes it particularly necessary to jointly administer M1 with a PD-L1 antibody.

The administration of OVs is rapidly shifting from intratumoral to intravenous delivery. For multifarious reasons (viral size, manufacturing-related concerns, and a high seroprevalence), most OVs are administered intratumorally, which limits the application of OVs to only easily accessible tumor sites. We show that intravenous injection of M1 plays therapeutic roles in subcutaneous melanoma and prostate cancer and may even pass through the blood-brain barrier to eradicate in situ brain gliomas. Intravenous administration could further extend the translatability of the M1 virus to nonsuperficial tumors and metastatic foci. We previously reported that M1 is nonpathogenic in normal rats, tumor-bearing mice, and nonhuman primates after multiple rounds of repeated i.v. injections^42,43^. The feasibility and safety of intravenous injection enhance the potential clinical value of M1.

Broadly speaking, the increasing understanding of M1 as a novel immunomodulatory agent and its multimodal antitumor mechanisms within the TME, coupled with observations of profound synergy with other cancer immunotherapies such as ICB, is creating renewed excitement for OVs in cancer immunotherapy.

## Supplementary information

Supplementary Figure Legends

Supplementary Fig 1

Supplementary Fig 2

Supplementary Fig 3

Supplementary Fig 4

Supplementary Fig 5

Supplementary Fig 6

Supplementary Fig 7

## Data Availability

Gene expression data have been deposited in the GEO repository under the accession number GSE152451.

## References

[1] Topalian SL (2012). Safety, activity, and immune correlates of anti-PD-1 antibody in cancer. N. Engl. J. Med..

[2] Hatae R, Chamoto K (2016). Immune checkpoint inhibitors targeting programmed cell death-1 (PD-1) in cancer therapy. Rinsho Ketsueki.

[3] Hodi FS (2010). Improved survival with ipilimumab in patients with metastatic melanoma. N. Engl. J. Med..

[4] Leach DR, Krummel MF, Allison JP (1996). Enhancement of antitumor immunity by CTLA-4 blockade. Science.

[5] Grote HJ (2020). Programmed death-ligand 1 immunohistochemistry assay comparison studies in NSCLC: characterization of the 73-10 assay. J. Thorac Oncol..

[6] Sharma P, Hu-Lieskovan S, Wargo JA, Ribas A (2017). Primary, adaptive, and acquired resistance to cancer immunotherapy. Cell.

[7] Ji RR (2012). An immune-active tumor microenvironment favors clinical response to ipilimumab. Cancer Immunol. Immunother..

[8] Ritprajak P, Azuma M (2015). Intrinsic and extrinsic control of expression of the immunoregulatory molecule PD-L1 in epithelial cells and squamous cell carcinoma. Oral Oncol..

[9] Gajewski TF, Schreiber H, Fu YX (2013). Innate and adaptive immune cells in the tumor microenvironment. Nat. Immunol..

[10] Sharma P, Allison JP (2015). The future of immune checkpoint therapy. Science.

[11] Tumeh PC (2014). PD-1 blockade induces responses by inhibiting adaptive immune resistance. Nature.

[12] Herbst RS (2014). Predictive correlates of response to the anti-PD-L1 antibody MPDL3280A in cancer patients. Nature.

[13] Yang X, Shi J, Chen X, Jiang Y, Zhao H (2020). Efficacy of cabozantinib and nivolumab in treating hepatocellular carcinoma with RET amplification, high tumor mutational burden, and PD-L1 expression. Oncologist.

[14] Parato KA, Senger D, Forsyth PA, Bell JC (2005). Recent progress in the battle between oncolytic viruses and tumours. Nat. Rev. Cancer.

[15] Auer R, Bell JC (2012). Oncolytic viruses: smart therapeutics for smart cancers. Future Oncol..

[16] Lott JB (2012). Oncolytic viruses: a new paradigm for treatment of head and neck cancer. Oral. Surg. Oral. Med Oral. Pathol. Oral. Radiol..

[17] Lawler SE, Speranza MC, Cho CF, Chiocca EA (2017). Oncolytic viruses in cancer treatment: a review. JAMA Oncol..

[18] Alemany R, Cascallo M (2009). Oncolytic viruses from the perspective of the immune system. Future Microbiol..

[19] Bell J (2014). Oncolytic viruses: immune or cytolytic therapy?. Mol. Ther..

[20] Chaurasiya S, Chen NG, Fong Y (2018). Oncolytic viruses and immunity. Curr. Opin. Immunol..

[21] Bommareddy PK, Patel A, Hossain S, Kaufman HL (2017). Talimogene laherparepvec (T-VEC) and other oncolytic viruses for the treatment of melanoma. Am. J. Clin. Dermatol..

[22] Burke J, Nieva J, Borad MJ, Breitbach CJ (2015). Oncolytic viruses: perspectives on clinical development. Curr. Opin. Virol..

[23] de Vries CR, Kaufman HL, Lattime EC (2015). Oncolytic viruses: focusing on the tumor microenvironment. Cancer Gene Ther..

[24] Atherton MJ, Lichty BD (2013). Evolution of oncolytic viruses: novel strategies for cancer treatment. Immunotherapy.

[25] Hu J, Cai XF, Yan G (2009). Alphavirus M1 induces apoptosis of malignant glioma cells via downregulation and nucleolar translocation of p21WAF1/CIP1 protein. Cell Cycle.

[26] Lin Y (2014). Identification and characterization of alphavirus M1 as a selective oncolytic virus targeting ZAP-defective human cancers. Proc. Natl Acad. Sci. USA.

[27] Cai J (2017). Selective replication of oncolytic virus M1 results in a bystander killing effect that is potentiated by Smac mimetics. Proc. Natl Acad. Sci. USA.

[28] Liang J (2018). Inhibition of the mevalonate pathway enhances cancer cell oncolysis mediated by M1 virus. Nat. Commun..

[29] Li K (2016). A classical PKA inhibitor increases the oncolytic effect of M1 virus via activation of exchange protein directly activated by cAMP 1. Oncotarget.

[30] Li K (2016). Activation of cyclic adenosine monophosphate pathway increases the sensitivity of cancer cells to the oncolytic virus M1. Mol. Ther..

[31] Green DR, Ferguson T, Zitvogel L, Kroemer G (2009). Immunogenic and tolerogenic cell death. Nat. Rev. Immunol..

[32] Ito H (2006). Autophagic cell death of malignant glioma cells induced by a conditionally replicating adenovirus. J. Natl Cancer Inst..

[33] Tesniere A (2008). Immunogenic cancer cell death: a key-lock paradigm. Curr. Opin. Immunol..

[34] Martins I (2014). Molecular mechanisms of ATP secretion during immunogenic cell death. Cell Death Differ..

[35] Griffith JW, Sokol CL, Luster AD (2014). Chemokines and chemokine receptors: positioning cells for host defense and immunity. Annu. Rev. Immunol..

[36] Scherwitzl I (2018). Systemically administered sindbis virus in combination with immune checkpoint blockade induces curative anti-tumor immunity. Mol. Ther. Oncolytics.

[37] Mostafa, A. A. et al. Oncolytic reovirus and immune checkpoint inhibition as a novel immunotherapeutic strategy for breast cancer. *Cancers* **10**, 205 (2018). 10.3390/cancers10060205 PMC6025420 29914097

[38] Mandai M (2016). Dual faces of IFNgamma in cancer progression: a role of PD-L1 induction in the determination of pro- and antitumor immunity. Clin. Cancer Res..

[39] Su X, Zhang J, Fu C, Xiao M, Wang C (2020). Recurrent metastatic penile cancer patient with positive PD-L1 expression obtained significant benefit from immunotherapy: a case report and literature review. Onco. Targets Ther..

[40] Hellmann MD (2018). Tumor mutational burden and efficacy of nivolumab monotherapy and in combination with ipilimumab in small-cell lung cancer. Cancer Cell.

[41] Hellmann MD (2018). Nivolumab plus ipilimumab in lung cancer with a high tumor mutational burden. N. Engl. J. Med..

[42] Zhang H (2016). Naturally existing oncolytic virus M1 is nonpathogenic for the nonhuman primates after multiple rounds of repeated intravenous injections. Hum. Gene Ther..

[43] Cai, J. et al. Systematic characterization of the biodistribution of the oncolytic virus M1. *Hum. Gene Ther.* **31**, 1203–1213 (2020). 10.1089/hum.2020.114 32829653

