# Supplementary material for: Intravenous injection of the oncolytic virus M1 awakens antitumor T cells and overcomes resistance to checkpoint blockade
Source: Cell Death Dis. 2020 Dec 12;11(12):1062. doi: 10.1038/s41419-020-03285-0 (PMC7733593; doi:10.1038/s41419-020-03285-0)
Supplement: Supplementary file 1 — Supplementary Figure Legends [file 41419_2020_3285_MOESM1_ESM.docx]

**Supplementary Information**

**Fig. S1. The efficacy and security of M1 virotherapy. (A)**, The RM-1 tumor size of mice treated with or without M1 virotherapy. **(B-D)**, The curves for weight changes are shown. The *p*-value was determined by repeated measures ANOVA.

**Fig. S2. The apoptosis pathway is upregulated and the DNA repair pathway is downregulated in M1-treated tumors. (A)** and **(B)**, C57BL/6J mice were implanted subcutaneously in the right flank with B16F10 cells on day 0 and treated intravenously with ctrl (n=4) or M1 (n=4) (1x10^7^ pfu) once per day on days 6-10. The tumors were harvested on day 12, total RNA was extracted, and RNA sequencing was then performed. GSEA results for apoptosis **(A)** and the DNA repair pathway **(B)** are shown.

**Fig. S3. UV irradiation eliminates infection and oncolysis by M1. (A)** and **(B)**, B16F10 cells were infected with M1-GFP (10 MOI) or UV-inactivated M1-GFP (10 MOI) for 24 h, expression of GFP was monitored **(A)**, and cell viability was measured **(B)**. Scale bar, 50 μm. n=4. The *p*-value was determined by one-way ANOVA with Dunnett’s test for pairwise comparisons.

**Fig. S4. B16F10 cells do not release IL-12, TNF-α or IL-1β after M1 infection. (A-C)**, B16F10 cells were infected with M1 (5MOI), and IL-12, TNF-α and IL-1β concentrations in supernatants were detected by ELISA two days later. n=3. The *p*-value was determined by a two-tailed Student’s t-test.

**Fig. S5. No viral particles were detected in lymphocytes isolated from mice receiving M1 virotherapy. (A)**, Lymphocytes isolated from B16F10 tumor tissue samples (TILs), tumor-draining lymph nodes (TDLN) and the spleen were cultured ex vivo, and supernatants were collected to detect viral titers via the TCID50 method. A virus solution with a known titer (1.5x10^6^ pfu/ml) served as a positive control.

**Fig. S6. The efficiency and specificity of anti-CD4 and anti-CD8 depletion antibodies. (A-C)**, Flow cytometry analysis confirmed the specific loss of CD4^+^ or CD8^+^ T cells in the TME **(A)**, spleen **(B)** and peripheral blood **(C)** with the indicated depletion antibody. Representative data from 3-4 mice are shown.

**Fig. S7. There is no weight loss during combined therapy with the M1 virus and PD-L1 antibody****. (A)** and **(B)**, Tumor models were established and M1 virotherapy was conducted as shown in Fig. 5C and D. The curve for weight changes is shown. The *p*-value was determined by repeated measures ANOVA.
