# Supplementary figures and images for: Intravenous injection of the oncolytic virus M1 awakens antitumor T cells and overcomes resistance to checkpoint blockade
Source: Cell Death Dis. 2020 Dec 12;11(12):1062. doi: 10.1038/s41419-020-03285-0 (PMC7733593; doi:10.1038/s41419-020-03285-0)

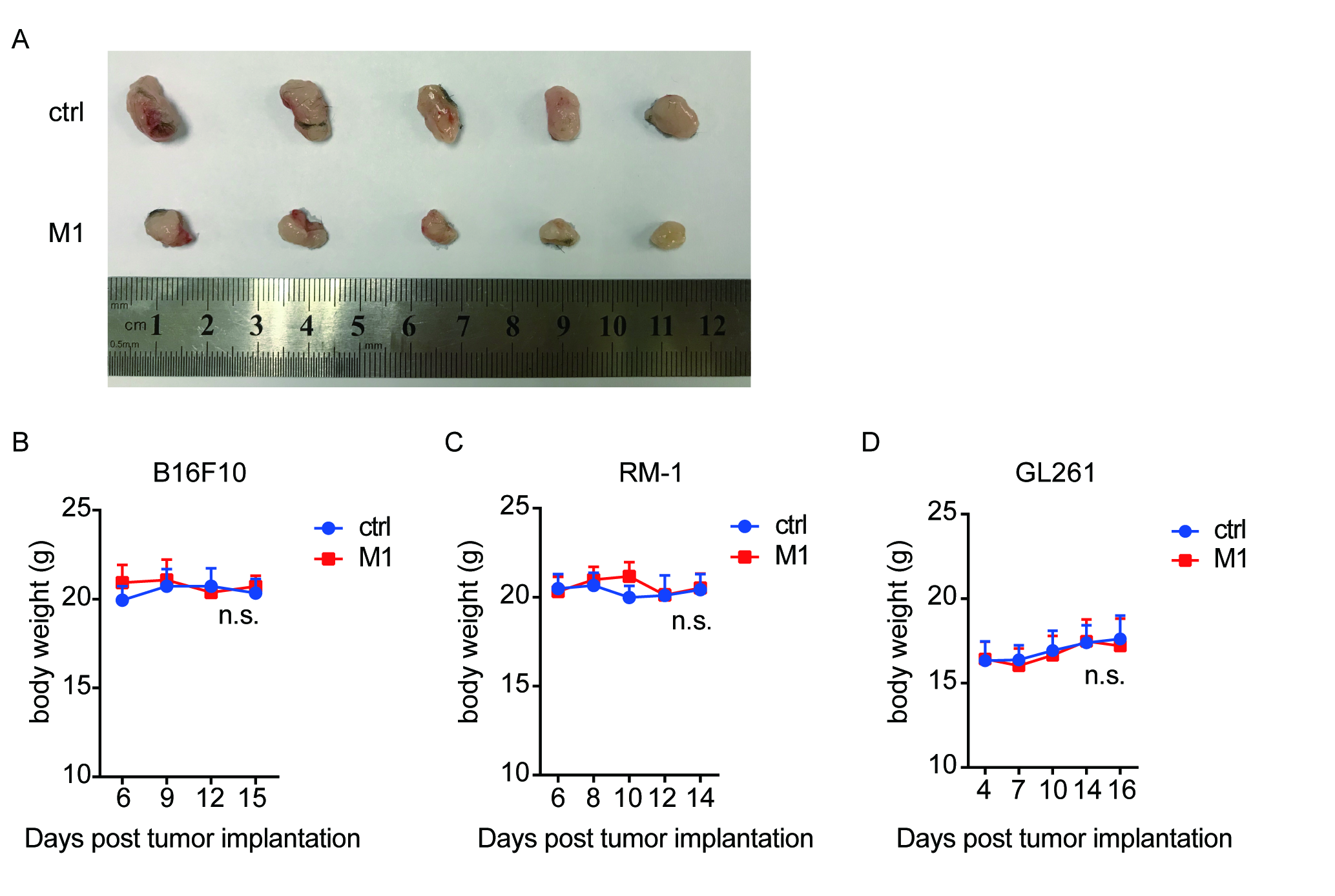

Supplement: Supplementary file 2 — Supplementary Fig 1 [file 41419_2020_3285_MOESM2_ESM.tif]

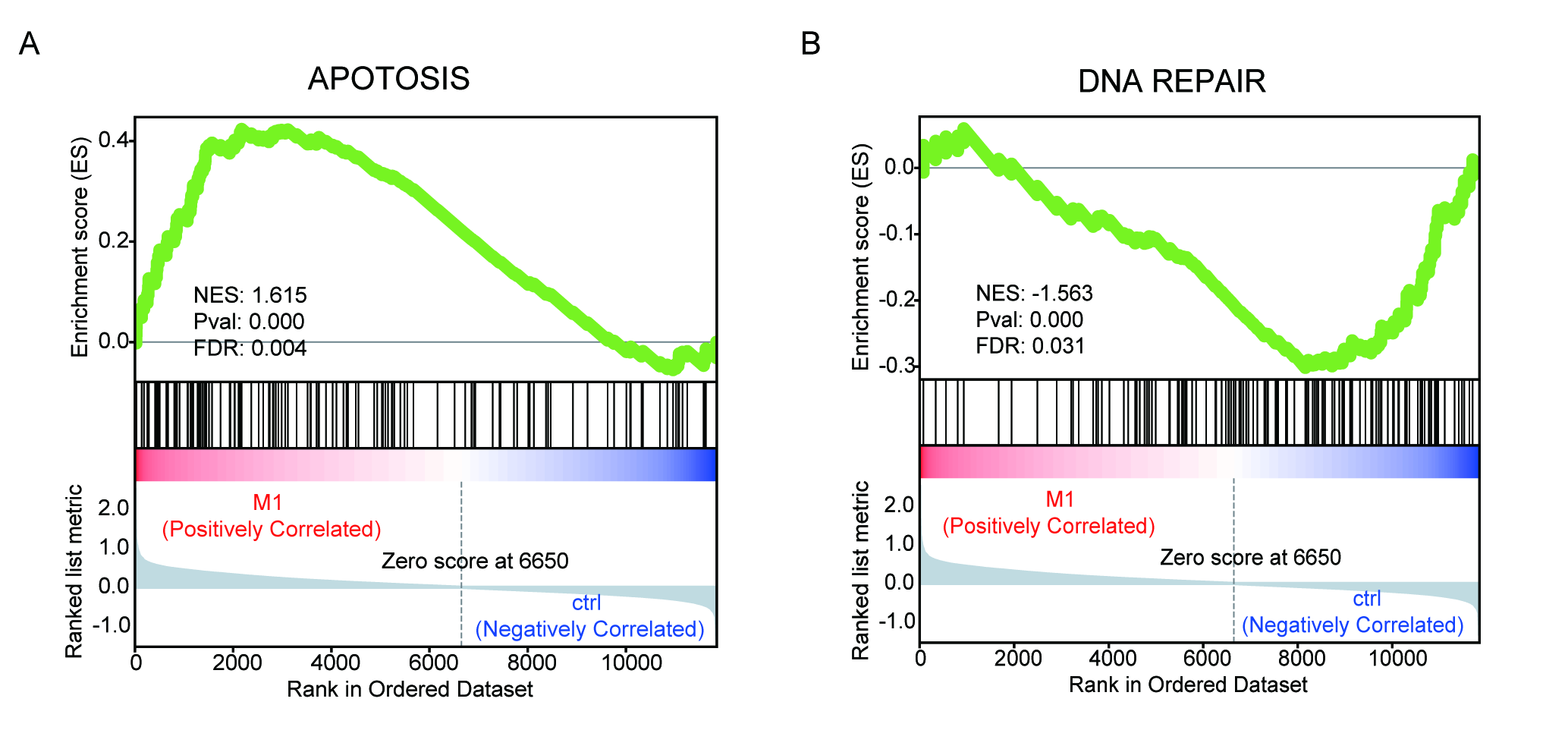

Supplement: Supplementary file 3 — Supplementary Fig 2 [file 41419_2020_3285_MOESM3_ESM.tif]

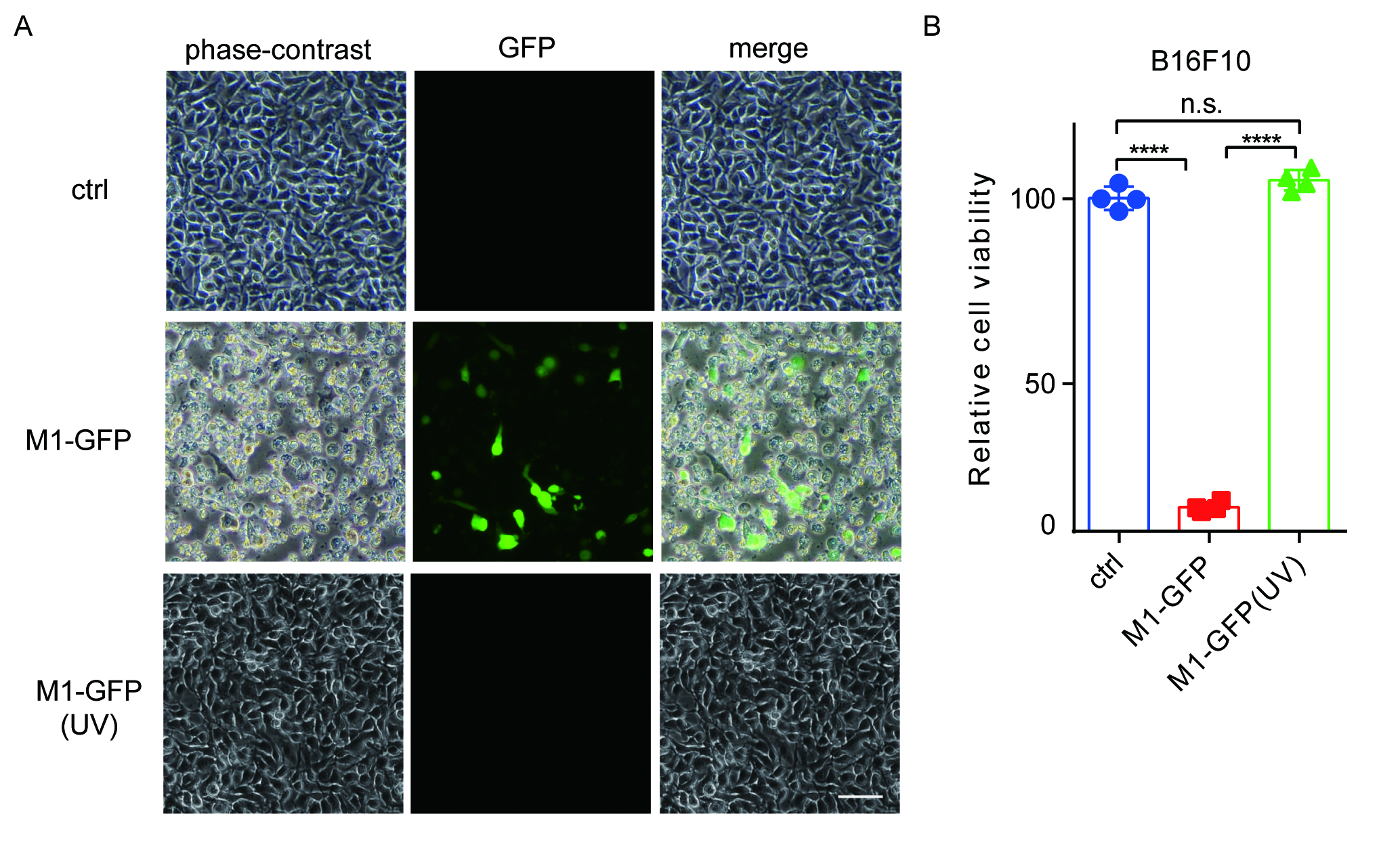

Supplement: Supplementary file 4 — Supplementary Fig 3 [file 41419_2020_3285_MOESM4_ESM.tif]

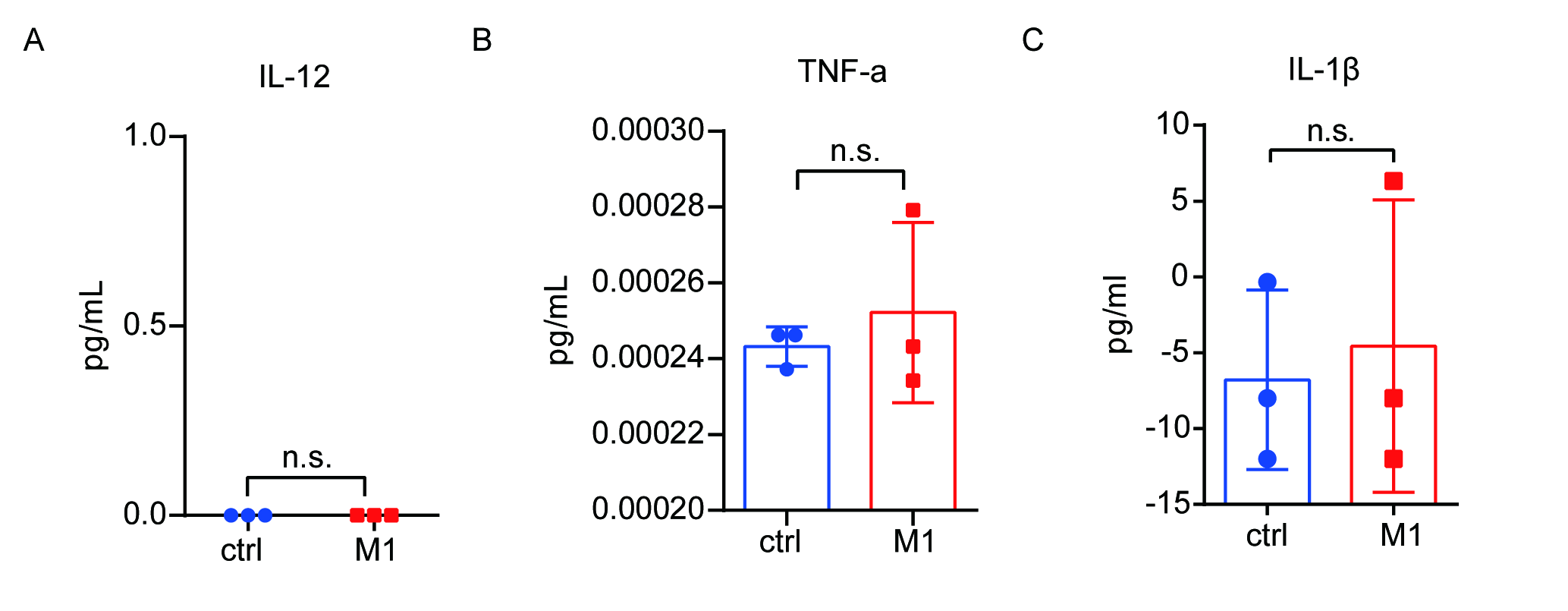

Supplement: Supplementary file 5 — Supplementary Fig 4 [file 41419_2020_3285_MOESM5_ESM.tif]

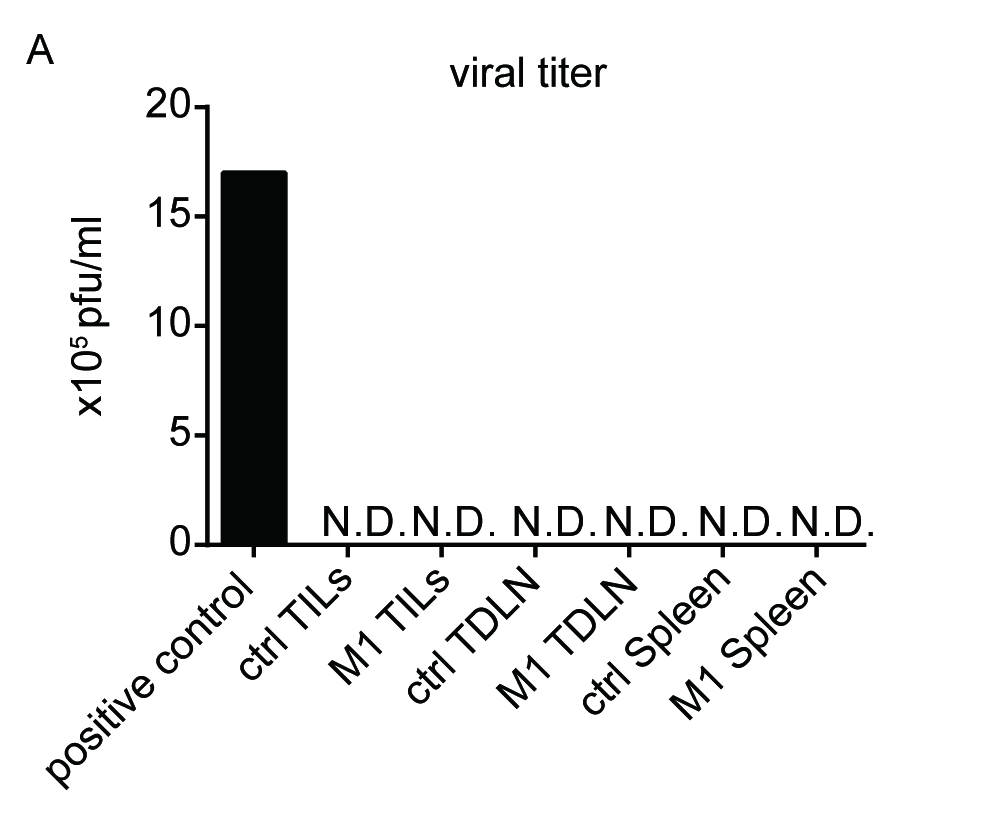

Supplement: Supplementary file 6 — Supplementary Fig 5 [file 41419_2020_3285_MOESM6_ESM.tif]

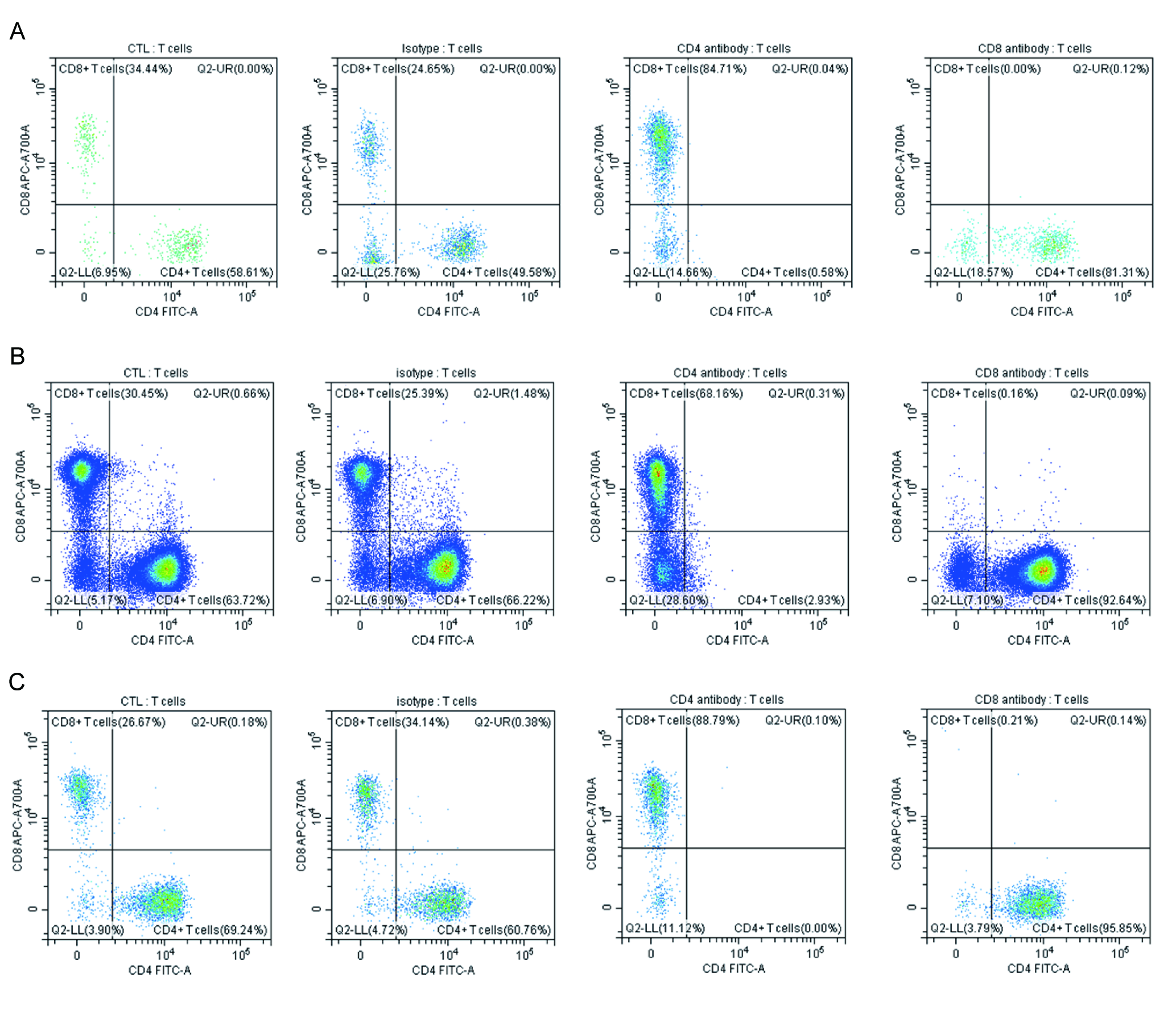

Supplement: Supplementary file 7 — Supplementary Fig 6 [file 41419_2020_3285_MOESM7_ESM.tif]

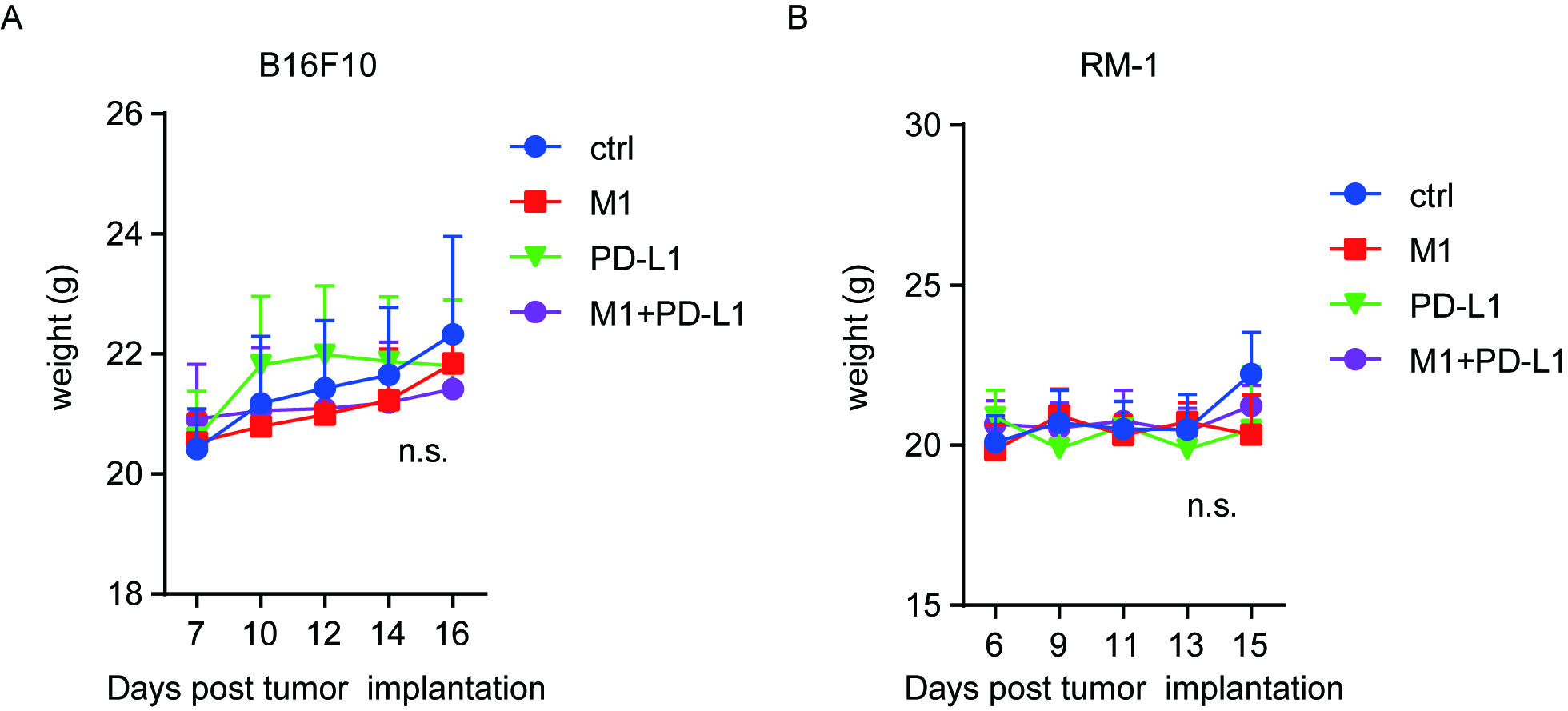

Supplement: Supplementary file 8 — Supplementary Fig 7 [file 41419_2020_3285_MOESM8_ESM.tif]
